# The nosological status of unipolar mania and hypomania within UK Biobank according to objective and subjective measures of diurnal rest and activity

**DOI:** 10.1111/bdi.13237

**Published:** 2022-06-16

**Authors:** Natasha Sangha, Laura Lyall, Cathy Wyse, Breda Cullen, Heather C. Whalley, Daniel J. Smith

**Affiliations:** ^1^ Mental Health and Wellbeing, Institute of Health and Wellbeing University of Glasgow Glasgow UK; ^2^ Department of Biology Maynooth University Kildare Ireland; ^3^ Centre for Clinical Brain Sciences, Division of Psychiatry University of Edinburgh Edinburgh UK

**Keywords:** accelerometery, bipolar disorder, circadian rhythms, UK Biobank, unipolar mania

## Abstract

**Background:**

There is uncertainty whether unipolar mania is a discrete sub‐type of bipolar disorder. Disrupted rest/activity rhythms are a key feature of bipolar disorder (BD) but have not been well characterised in unipolar mania/hypomania (UM). We compared subjective and objective rest/activity patterns, demographic and mental health outcomes across BD, UM and control groups.

**Methods:**

UK residents aged 37–73 years were recruited into UK Biobank from 2006 to 2010. BD, UM and control groups were identified via a mental health questionnaire. Demographic, mental health and subjective sleep outcomes were self‐reported. Accelerometery data were available for a subset of participants, and objective measures of sleep and activity were derived.

**Results:**

A greater proportion of males met UM criteria, and more females were in the BD group. Both BD and UM groups had poor mental health outcomes vs. controls. Objectively measured activity differed between all three groups: UM had highest levels of activity and BD lowest. The UM group had shorter sleep duration compared to controls. Subjective rest/activity measures showed that both mood disorder groups (compared to controls) had later chronotype preference, more disturbed sleep and increased difficulty getting up in the morning. However, the UM group were more likely to report an early chronotype compared to BD and control groups.

**Conclusions:**

BD and UM share features in common, but key differences support the proposition that UM may be a distinct and more clinically homogenous disorder. UM was characterised by a higher proportion of males, early chronotype, increased activity and shorter sleep duration.

## 1 INTRODUCTION

There is a longstanding debate about individuals who experience episodes of mania or hypomania in the absence of depressive episodes (so‐called unipolar mania, UM) and whether they should be considered as nosologically distinct from individuals with bipolar disorder (BD). Currently, both DSM‐5 and ICD‐11 incorporate UM as part of bipolar‐I disorder.^1^ It has been suggested that including UM within BD hinders research on the pathophysiology of mania and increases heterogeneity within BD research.^2^

Studies comparing characteristics of UM groups to BD groups are relatively rare, but some work suggests that there may be differences in both demographic and lifestyle factors, as well as mental health outcomes. For example, the sex distribution in BD is approximately equal but UM may be more common in males.^3^, ^4^ Individuals with UM tend to experience more manic episodes than those with BD but have similar treatment and self‐harm characteristics.^5^ People with UM may also have less social disability and higher scores for hyperthymic temperament.^6^ In contrast, some studies have found UM groups to be at higher risk of hospitalisation, greater use of medications and worse overall functioning compared to BD.^7^ BD tends to be associated with a higher risk of comorbid Attention Deficit Hyperactivity Disorder (ADHD) and anxiety disorders than UM.^3^, ^7^ Overall, low participant numbers in these studies make it difficult to identify consistent and objective biological and/or phenotypic markers that might differentiate between UM and BD.

One area that may provide new insights on biological or phenotypic markers is sleep and circadian rhythm function. A large body of research has highlighted associations between disturbed circadian rhythms and mood disorders, particularly BD.^8^, ^9^, ^10^, ^11^ Morningness‐eveningness preference (chronotype) is a behavioural phenotype for circadian rhythm timing which has good reproducibility over time,^12^, ^13^ although subjective measures such as chronotype are more prone to reporting bias than objective measures such as actigraphy. Although findings are mixed overall, multiple studies have identified chronotype differences between BD and control groups, with later chronotype (a preference for evening activity) more commonly observed in people with BD, even before the onset of illness.^14^, ^15^ The relationship between chronotype and UM has not yet been investigated in detail but one study found no difference in chronotype preference between sub‐threshold mania and controls.^16^ Mania is more typically examined in the context of BD where published findings have shown that chronotype differences exist regardless of polarity or mood state in BD,^14^ and that late chronotype is associated with a more depressive course of BD.^17^

Actigraphy can provide an objective and naturalistic approach to measuring diurnal patterns of rest and activity. A small activity monitor, usually worn on a wrist, measures acceleration, frequency and direction of movement.^13^ Actigraph studies have identified aberrant rhythms of rest and activity in BD.^14^, ^18^ This includes lower activity levels throughout the day, longer sleep duration (but more disturbed sleep), and a less stable daily rhythm compared to controls. No studies to date have explicitly compared actigraphy measures in UM versus BD, but there is inconsistent evidence of associations between manic episodes and changes in objectively measured sleep duration, sleep quality and activity levels (both increases and decreases have been reported).^19^, ^20^, ^21^, ^22^, ^23^

We set out to test for similarities and differences between UM, BD, and a non‐mood disordered comparison group within the large UK Biobank cohort, making use of a broad range of demographic, lifestyle and mental health outcomes and with a particular focus on objective and subjective measures of diurnal patterns of activity and sleep. Our primary hypothesis was that individuals with UM would have a different profile of sleep and timing of diurnal rest/activity compared to individuals with BD.

## 2 METHODS

### 2.1 Participants

We used data from UK Biobank which comprises of a range of data on health, lifestyle, demographic and physical characteristics from over 502,000 UK residents. These tests and questionnaires were administered at testing centres across the UK from 2006 onwards and included questionnaires relating to mental health, and (for a subset of participants, *N* = 103,617) data from wrist‐worn accelerometers. Participants who self‐reported severe neurological diagnoses, brain cancer/injury, personality disorder, psychosis, schizophrenia, sleep apnoea/insomnia, or a main job that involved shift‐work were excluded from the analysis (*N* = 29,522).

### 2.2 Probable mood disorder criteria

Participants were provided with a touchscreen mental health questionnaire within which there were five mania‐specific questions (this questionnaire was introduced part way through UK Biobank recruitment and so was only administered to a subset of participants, *N* = 214,576). Two of these questions identified whether a participant had a period of elevated mood or irritability lasting at least two days, and participants only answered the remaining mania questions if they answered yes to one of these questions. The remaining three questions assessed symptoms experienced during this time, duration of the episode, and how problematic the episode was. This questionnaire also assessed depressed mood, firstly by identifying whether the participant had experienced depressed feelings or anhedonia lasting 2+ weeks and, if yes, assessing additional symptoms, number of episodes and how problematic the episode had been. In our analysis, participants were identified as having probable UM if they answered yes to being 'irritable' or ‘hyper’ for two days or more *and* had experienced 3 or more manic symptoms. Participants were considered to have probable BD if they met UM criteria and additionally met criteria for single or recurring major depression (yes to 2 or more weeks of depressed feelings or anhedonia, 5 or more depressive symptoms and a health professional had been consulted about these symptoms). These criteria resemble the DSM5 criteria as closely as possible within the limitations of the questions available within the UK Biobank cohort.

A subset of UK Biobank participants opted in for completion of a specific mental health follow‐up questionnaire completed online in 2016–2017 (*N* = 157,317). The mania section of this questionnaire was structured similarly to the baseline touchscreen mental health questionnaire, although the multiple‐choice options for symptoms and duration questions were slightly different. The online symptoms assessment had eight symptoms whereas the touchscreen version only had four symptoms. The duration options included ‘less than 24 h’, ‘more than one day but less than a week’, and ‘a week or more’ (the touchscreen duration included ‘less than a week’, ‘less than a week but more than two days’, and ‘a week or more’). Due to the additional level of detail, if a participant had completed this more comprehensive online questionnaire, the mood disorder criteria were applied from this assessment rather than from the touchscreen questionnaire.

### 2.3 Measurement of activity and sleep outcomes

Participants who opted into the UK Biobank accelerometer sub‐study were provided with an AX3 triaxial accelerometer (Axivity, Newcastle upon Tyne, UK) and asked to wear this on their dominant wrist for 7 days. Data collection took place from 2013 to 2016. The UK Biobank Accelerometer Expert Working Group conducted data pre‐processing and provided acceleration averages which were then used to calculate the following variables (further details are available at http://biobank.ctsu.ox.ac.uk/crystal/docs/PhysicalActivityMonitor.pdf).

*Average Acceleration (AA)* is the average level of activity over the full measurement period.^24^ *Relative Amplitude (RA)* is the relative difference between the most active 10‐hour period and least active 5‐hour period of a given day, calculated as an average across all days of available data. Lower RA values suggest disturbed sleep and/or lower levels of daytime activity. *Interdaily Stability (IS)* indicates the level of coupling of activity levels to 24‐hour daily patterns. Higher values suggest a regular daily rhythm whereas lower values indicate more variation in wake‐up times or activity levels across various days. *Intradaily Variability (IV)* quantifies how fragmented the daily rhythm is, with higher values suggesting disturbed sleep or periods of inactivity during the daytime. Further information on the calculation of RA, IS and IV can be found here.^25^ *Mean Sleep Duration* is the number of hours spent sleeping within the sleep window (i.e. between going to bed and getting out of bed), averaged across all days of wear. *Mean Sleep Efficiency* quantifies the amount of time spent sleeping as a proportion of the total sleep window, with higher values suggesting less disturbed sleep. Further information on the calculation of mean sleep duration and mean sleep efficiency can be found here.^26^ In total 25,388 participants had complete accelerometer data which passed quality control and met the criteria for either probable BD, probable UM, or no mood disorder.

As well as the above objective measures, participants at recruitment completed questionnaires that included questions relating to sleep. This included self‐reported average sleep duration, level of difficulty getting up in the morning (lower values indicated more difficulty), sleeplessness (higher values indicated more sleeplessness), and chronotype (higher values indicated more eveningness).

### 2.4 Measurement of mental health and psychological outcomes

The mental health follow‐up questionnaire included questions relating to happiness, of which two were considered: ‘In general how happy are you?’ and ‘In general how happy are you with your health?’, both of which had options of extremely, very or moderately happy/unhappy. Within this questionnaire participants also reported whether they had ever self‐harmed and if they had ever experienced anxiety (“a period lasting one month or longer when most of the time you felt worried, tense, or anxious”).

Within the touchscreen mental health questionnaire, participants reported whether they considered themselves to be risk‐takers. A neuroticism score was also calculated based upon the answers to twelve questions that cover domains of neurotic behaviour.^27^

### 2.5 Additional measures

During the baseline assessment visits participants provided demographic and lifestyle information including age, sex, ethnicity, educational attainment, smoking status and alcohol intake. Postcode of residence at the time of the assessment was used to derive Townsend deprivation scores. Body‐mass index (BMI) was calculated from measurements of height and weight taken at the time of assessment. Regular prescription medication was recorded by a trained nurse during the verbal interview section at the assessment centre and this was summarised into a categorical variable representing psychotropic medication use if the medication was any of those listed in Appendix S1.

### 2.6 Statistical analysis

Group differences for each variable of interest across each of the mental health groups (BD, UM, controls) were examined using individual multivariate logistic regression models. Three group comparisons were performed for each variable of interest: BD vs UM; UM vs control; and BD vs control.

Continuous relative amplitude, interdaily stability, average acceleration and sleep efficiency were inverted so that an increased score reflected a more negative outcome, for consistency with all other comparisons. These continuous variables, along with intradaily variability, were divided into quintiles due to their narrow ranges.

Both objective and subjective sleep duration were categorised into short (less than 7 h), regular (between 7 and 9 h) and long (more than 9 h), with regular as the reference category.^28^ Chronotype was condensed into three categories: early (‘definitely a morning person’), intermediate (‘more morning than evening’ and ‘more evening than morning’) and late (‘definitely an evening person’), with intermediate as the reference category. Participants who chose not to answer or answered ‘do not know’ were excluded.

Objective and subjective sleep duration were compared in a follow‐up analysis to assess whether there were group differences in overestimating or underestimating sleep duration. These measures are not directly comparable as participants were asked to include daytime napping in the subjective sleep duration question, but napping is not included within the objective sleep duration estimate. For this reason participants who reported regular napping in the multiple‐choice questionnaire were excluded. For the remaining participants subjective sleep duration was subtracted from objective sleep duration (rounded to the nearest hour) to estimate the objective/subjective sleep duration difference. If the sleep duration difference was 0 this was categorised as ‘accurate’. If the difference was greater than 0 this was categorised as an ‘overestimation’, and less than 0 was categorised as an ‘underestimation’.

General happiness and happiness with health were both condensed into two categories: happy (‘extremely happy’, ‘very happy’, ‘moderately happy’) and unhappy (‘extremely unhappy’, ‘very unhappy’, ‘moderately unhappy’). Getting up in the morning was also condensed into a binary category of not difficult (‘fairly easy’ and ‘very easy’) and difficult (‘not at all easy’ and ‘not very easy’) with not difficult as the reference category. Participants who chose not to answer or answered ‘Do not know’ were excluded.

For each of these comparisons the multivariate logistic regression model was both partly adjusted and fully adjusted. The partly adjusted models included age, sex, Townsend deprivation score, education level and ethnicity as covariates. The season in which the accelerometer was worn as a covariate was also included in the partly adjusted model for objectively measured variables of interest. The fully adjusted models additionally included BMI, smoking status, alcohol status and psychotropic medication status. Group comparisons for lifestyle and demographic variables were assessed with one‐way ANOVA for continuous numeric variables and Pearson's chi‐squared test for categorical variables. All statistical analyses were performed using R version 3.6.1 (R Core Team 2019. R: A language and environment for statistical computing. R Foundation for Statistical Computing, Vienna, Austria.). False Discovery Rate (FDR) correction was applied to the probability values of the fully adjusted models. The acceptable FDR was defined as <0.05.

## 3 RESULTS

### 3.1 Demographic/Lifestyle comparisons

All assessed demographic variables varied significantly between groups (Table 1), confirming their importance as covariates within the subsequent models. UM participants were on average younger than control participants but older than the BD group. The UM group had a higher proportion of male participants, whereas the BD group had more female participants. The control group had significantly lower Townsend deprivation scores (indicating greater affluence) and the UM group were more affluent than the BD group. The BD group were more likely to have a higher BMI than both the UM and control groups, with the UM group having a higher BMI than the control group. Both the UM and BD groups had a higher rate of current or past smoking compared to the control group, with the BD group twice as likely to be current smokers as the UM group. The BD group were more likely to have given up drinking alcohol than the UM and control groups.

**TABLE 1:** Descriptive statistics of demographic and lifestyle variables

	Mean (SD) / Percentage (*N*)			Test statistic	*p* value	Effect size
	BD	UM	Control			
Age	52.11 (7.31)	54.05 (7.76)	57.06 (7.56)	1030	<0.001	0.031
Sex	–	–	–	437.99	<0.001	0.083
Female	61.66 (2978)	39.12 (470)	46.65 (27,166)	–	–	–
Male	38.34 (1852)	60.83 (730)	53.35 (31,072)	–	–	–
Townsend Score	−1.02 (3.16)	−1.71 (2.82)	−2.00 (2.67)	297.6	<0.001	0.009
Education	–	–	–	87.63	<0.001	0.026
Incomplete	6.15 (295)	3.60 (43)	8.16 (4703)	–	–	–
Compulsory	15.05 (722)	12.13 (145)	14.45 (8336)	–	–	–
Continued	7.36 (353)	6.95 (83)	6.07 (3498)	–	–	–
College	29.14 (1398)	28.28 (338)	27.07 (15,612)	–	–	–
University	42.31 (2030)	49.04 (586)	44.25 (25,521)	–	–	–
Ethnicity	–	–	–	77.01	<0.001	0.025
White	96.30 (4637)	95.82 (1146)	97.06 (56,374)	–	–	–
Mixed	1.14 (55)	0.59 (7)	0.37 (214)	–	–	–
Asian or Asian British	0.98 (47)	1.42 (17)	0.98 (569)	–	–	–
Black or Black British	0.85 (41)	0.92 (11)	0.84 (489)	–	–	–
Chinese	0.17 (8)	0.17 (2)	0.27 (159)	–	–	–
Other Ethnic Group	0.56 (27)	1.09 (13)	0.48 (277)	–	–	–
BMI	27.81 (5.19)	27.25 (4.36)	26.53 (4.14)	217.3	<0.001	0.007
BMI (> = 18.5 & < 30)	*25.33 (2.68)*	*25.58 (2.57)*	*25.16 (2.59)*	*18.21*	*<0.001*	*0.001*
Smoking Status	–	–	–	764.26	<0.001	0.077
Never	48.18 (2321)	55.63 (667)	61.46 (35,714)	–	–	–
Previous	37.16 (1790)	37.03 (444)	33.05 (19,206)	–	–	–
Current	14.66 (706)	7.34 (88)	5.50 (3194)	–	–	–
Alcohol Status	–	–	–	287.36	<0.001	0.047
Never	2.45 (118)	2.08 (25)	3.01 (1753)	–	–	–
Previous	5.59 (270)	2.58 (31)	1.91 (1114)	–	–	–
Current	91.96 (4438)	95.33 (1144)	95.07 (55,330)	–	–	–
Any Psychotropic Medication	–	–	–	906.47	<0.001	0.119
Yes	3.77 (182)	0.33 (4)	0.33 (191)	–	–	–
No	96.23 (4648)	99.67 (1196)	99.67 (58,047)	–	–	–

### 3.2 Mental health and psychological comparisons

**FIGURE 1:**
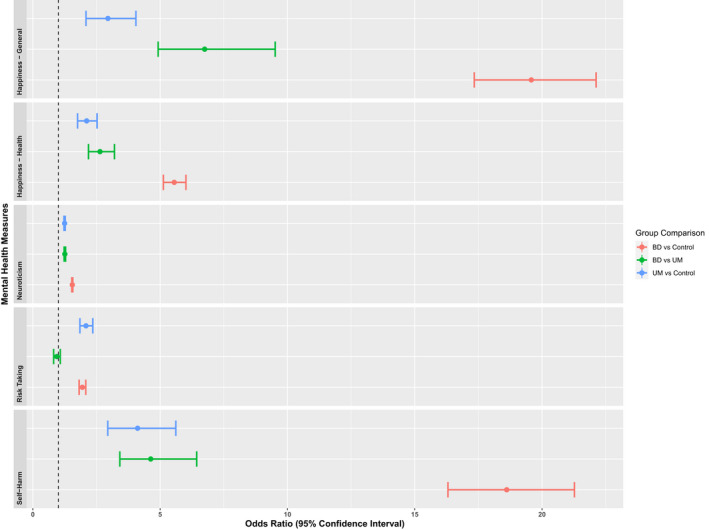
Group comparisons of mental health and psychological variables. Odds Ratios and their 95% Confidence Intervals for measures related to mental health and psychological outcomes. Anxiety is not included in this figure due to the difference in scale. Higher odds ratios reflect a more negative outcome.

As expected, both mood disorder groups had poor mental health/psychological outcomes compared to the control group (Figure 1; Table 2). This included greater levels of reported anxiety (BD vs Control OR = 47.97, 95% CI = 44.03, 52.30; UM vs Control OR = 3.01, 95% CI = 2.54, 3.56; BD vs UM OR = 15.47, 95% CI = 12.93, 18.59). The anxiety results are not included within Figure 1 due to scale differences ‐ the ORs were very high due to much higher proportions of BD and UM reporting anxiety compared to controls (Table 2). For most measures, the BD group were more likely to report a negative outcome, followed by the UM group. The only exception to this was risk‐taking behaviour, where both the UM and BD groups were more likely to declare themselves risk‐takers than controls (but were not different from each other).

**TABLE 2:** Descriptive statistics of mental health, sleep and activity

Measure	BD		UM		Control	
	Sample Size	Mean (SD) / % (*N*)	Sample Size	Mean (SD) / % (*N*)	Sample Size	Mean (SD) / % (*N*)
General happiness	4632	80.03% (3707)	1151	96.52% (1111)	51,651	98.95% (51109)
Happiness with health	4691	70.33% (3299)	1150	87.22% (1003)	51,667	93.97% (48552)
Neuroticism	3989	6.95 (3.27)	1038	4.35 (3.13)	49,685	2.54 (2.55)
Risk taking	4629	39.34% (1821)	1158	43.52% (504)	55,974	24.65% (13798)
Self‐harm	4674	17.86% (835)	1151	3.82% (44)	51,685	0.79% (406)
Anxiety	4210	74.16% (3122)	1123	15.41% (173)	51,100	5.71% (2917)
Relative amplitude (RA)	1889	0.86 (0.07)	495	0.86 (0.07)	23,004	0.87 (0.06)
Average acceleration (AA)	1889	28.40 (8.72)	495	29.29 (8.22)	23,004	28.32 (8.23)
Interdaily stability (IS)	1889	0.53 (0.13)	495	0.53 (0.14)	23,004	0.54 (0.13)
Intradaily variability (IV)	1889	0.92 (0.24)	495	0.92 (0.27)	23,004	0.93 (0.25)
Sleep efficiency	1889	0.76 (0.08)	495	0.75 (0.08)	23,004	0.76 (0.07)
Objective sleep duration	1889	–	495	–	23,004	–
< 7 h (Short)	–	36.95% (698)	–	42.42% (210)	–	33.45% (7695)
7–9 h (Normal)	–	61.04% (1153)	–	57.17% (283)	–	65.05% (14964)
> 9 h (Long)	–	2.01% (38)	–	0.40% (2)	–	1.50% (345)
Subjective sleep duration	4771	–	1189	‐–	57,443	–
<7 h (Short)	–	29.78% (1421)	–	24.81% (295)	–	19.27% (11068)
7–9 h (Normal)	–	68.06% (3247)	–	74.26% (883)	–	80.01% (45960)
>9 h (Long)	–	2.16% (103)	–	0.93% (11)	–	0.72% (415)
Sleeplessness	4775	2.20 (0.69)	1191	2.02 (0.71)	57,486	1.89 (0.72)
Difficulty Getting up	4773	29.54% (1410)	1191	15.45% (184)	57,498	10.84% (6233)
Chronotype	4429	–	1105	–	50,918	–
Early	–	24.43% (1082)	–	29.86% (330)	–	26.91% (13704)
Intermediate	–	61.14% (2708)	–	59.46% (657)	–	65.76% (33483)
Late	–	14.43% (639)	–	10.68% (118)	–	7.33% (3731)

### 3.3 Objective activity and sleep assessments

**FIGURE 2:**
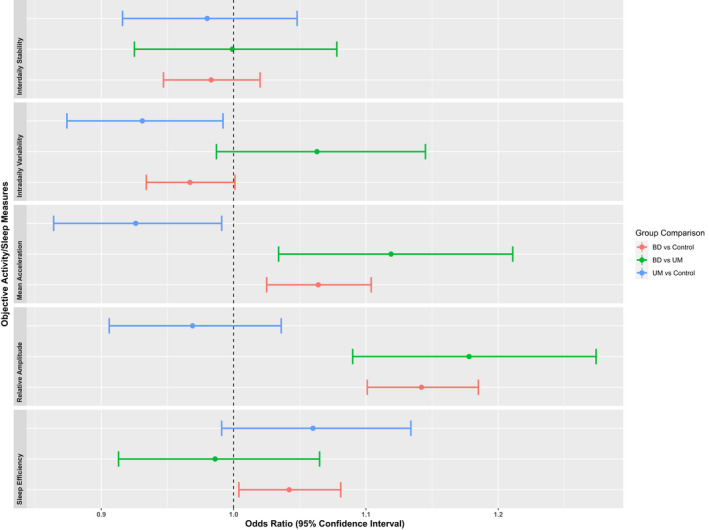
Group comparisons of objective measures of rest and activity. Odds Ratios and their 95% Confidence Intervals for objectively measured sleep and activity variable quintiles.

Figure 2 shows the odds ratios and confidence intervals for the objective measures of activity and sleep (AA, IS, IV, RA, and Sleep Efficiency quintiles). IS did not differ in any of the comparisons, suggesting that all three groups had a similar level of rhythm stability across the seven measured days. Both mood disorder groups had more defined activity and rest periods within each day compared to the control group, as shown by IV; however, this effect was only significant for the UM group. Despite better IV, the BD group had lower RA than both the control and UM groups, suggesting less differentiation between periods of sleep and activity in this group. Comparison of AA shows that the BD group had lower overall levels of activity than the control group, whilst the UM group had higher levels of activity than the control group. Both mood disorder groups also exhibited lower sleep efficiency (more disturbance during the sleep period) compared to the control group, although this difference was only significant for the BD group.

### 3.4 Objective and subjective sleep duration

**FIGURE 3:**
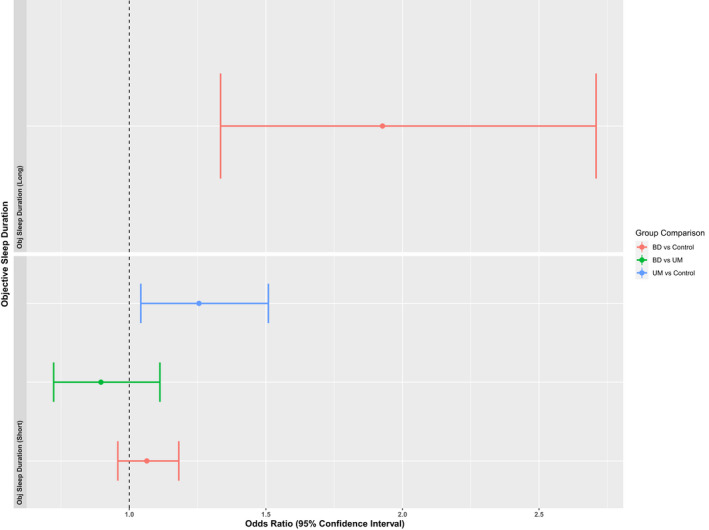
Group comparisons of objective sleep duration. Odds Ratios and their 95% Confidence Intervals for objectively measured sleep duration. Short sleep duration is defined as <7 h and long sleep duration is defined as >9 h. Groups are compared to a reference group that averaged 7–9 h of sleep over the period of accelerometer wear. Long sleep comparisons involving the UM group are not included due to small group numbers.

**FIGURE 4:**
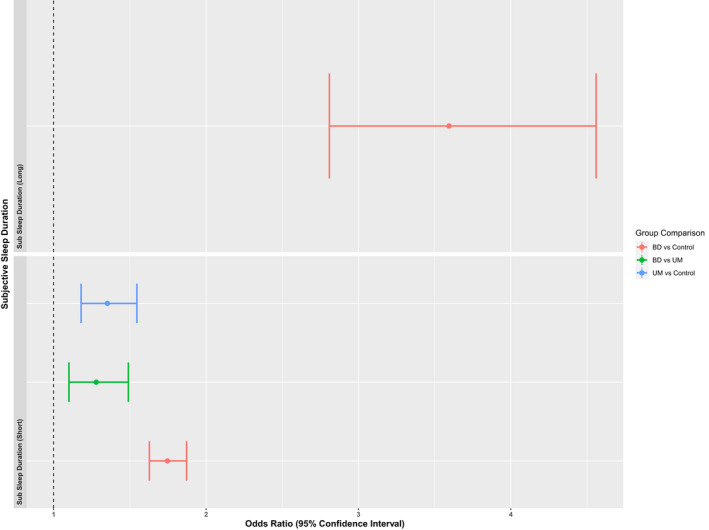
Group comparisons of subjective sleep duration. Odds Ratios and their 95% Confidence Intervals for subjectively measured sleep duration. Short sleep duration is defined as <7 h and long sleep duration is defined as >9 h. Groups are compared to a reference group that reported 7–9 h of sleep. Long sleep comparisons involving the UM group are not included due to small group numbers.

Figures 3 and 4 show the logistic regression results for objective and subjectively measured sleep duration. A very small number of participants in the UM group met criteria for long sleep duration (>9 h; objective sleep duration *N* = 2, subjective sleep duration *N* = 11), so Fishers Exact Test was used for those group comparisons instead of logistic regression (Supplementary Table S1).

Having a long sleep duration, as derived from the 7‐day accelerometer data, was more likely in the BD group than both UM and control groups. The UM group were more likely to have a shorter average sleep period (<7 h) than the control group.

As with the objective measure, BD was associated with longer subjective sleep duration. However, although only UM were associated with short sleep duration on the objective measure, both UM and BD were associated with short subjective sleep duration compared to controls. It is worth noting that these findings are not directly comparable as they were derived from different (but overlapping) populations.

In a follow‐up analysis, group differences in the accuracy of reported sleep duration were assessed. Table 3 summarises the differences in observed and subjectively estimated sleep duration within the UM, BD and control groups. The UM group were most likely to overestimate sleep duration and the BD group were most likely to underestimate sleep duration. As shown in the multinomial logistic regression analysis (Supplementary Table S2), the UM group had an increased likelihood of incorrect estimation (both under‐ and overestimation) compared to the control group, whereas the BD group only exhibited an increased risk of underestimation compared to controls.

**TABLE 3:** Group comparisons of the difference between objective and subjective sleep duration

	BD	UM	Control
Mean difference (Minutes)	17.3	5.8	7.2
95% Confidence interval	(12.3, 22.5)	(−2.8, 14.3)	(6.0, 8.4)
Test statistic	6.666	1.332	12.165
*p* value	<0.001	0.184	<0.001
*r* ^2^	0.09	0.219	0.156
Underestimated (%)	43.36	40.19	36.51
Accurate (%)	32.72	30.23	37.76
Overestimated (%)	23.92	29.58	25.73

*Note:* Test statistics are paired samples *t*‐values comparing the mean difference between objectively and subjectively measured sleep duration for each group. Subjective estimations are classed as “accurate” if they are equal to the objective sleep duration (derived from accelerometer wear data, rounded to the nearest hour).

### 3.5 Subjective activity and sleep comparisons

**FIGURE 5:**
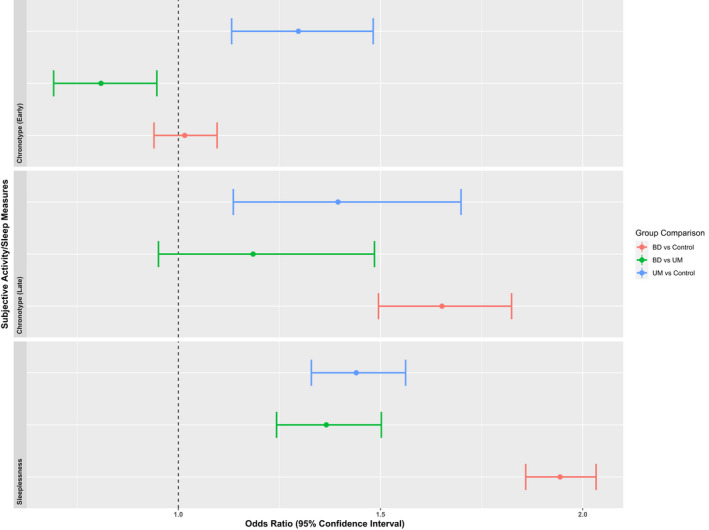
Group comparisons of the difference between subjective measures of activity and sleep. Odds Ratios and their 95% Confidence Intervals for self‐report measures of sleep and activity

The mood disorder groups reported more difficulties with sleep than the control group. As seen in Figure 5, both the BD and UM group were more likely to report disturbed sleep and difficulty getting up, but the BD group reported this to a greater extent than the UM group. This was also true for late chronotype, however early chronotype (a preference for activity in the morning) was reported more often in the UM group compared to both control and BD groups.

## 4 DISCUSSION

Categorical classifications are useful in clinical settings and account for a small number of changes to rest and activity rhythms when classifying bipolar disorders. However, the significant overlap between bipolar disorder categories and high heterogeneity within these groups has inspired interest in dimensional approaches to understanding these groups.^29^ Approaches such as the Hierarchical Taxonomy of Psychopathology (HiTOP) and Research Domain Criteria Initiative (RDoC) aim to address this issue by encouraging research into underlying biological and behavioural systems of psychopathology.^30^, ^31^ RDoC specifically identify ‘sleep‐wakefulness’ as of interest in psychiatric illness, and the findings above consider behavioural and self‐report aspects of this domain.

Overall, several of the findings described above are of interest with respect to similarities and differences between UM and BD in terms of mental health, wellbeing, sleep and activity characteristics. The UM group had a higher proportion of males than the BD group, in keeping with other reports.^3^, ^4^ It is well established that depression affects more females than males; however, it is also possible that the UM group may have experienced some sub‐threshold depressive symptoms.^32^ Other demographic characteristics also support previous findings, including higher levels of educational attainment in the UM group. Although a limitation of UK Biobank is the low heterogeneity in reported ethnicity, there was a greater proportion of ‘Asian or Asian British’ ethnicity reported within the UM group. This may be consistent with some reports that rates of UM and mania‐predominant BD are higher in South Asia.^33^, ^34^

Across all mental health and wellbeing measures, both BD and UM groups reported more negative outcomes compared to controls. Although the BD group generally had worse outcomes than the UM group, this was not true for risk‐taking where UM and BD groups were comparable.

Both mood disorder groups reported worse outcomes than controls on sleep and activity. As with the mental health outcomes, the BD group reported the worst outcomes, followed by the UM group. An exception to this was reported chronotype. Late chronotype (a preference for evening activity) was more likely in BD than healthy controls, consistent with previous findings.^16^, ^18^, ^35^ However, the UM group were more likely to report both early and late chronotypes. The literature suggests that late chronotype may be related to more severe depressive episodes and a reduction in manic episodes,^17^ and that episodes of mania are related to an advance in phase,^36^ which broadly supports the relationship between UM and early chronotype. Early chronotype preference may therefore be a useful distinguishing feature of UM.

There were differences in sleep duration between UM and BD. Sleep duration was measured both subjectively as part of a questionnaire, and objectively during the 7‐day accelerometer assessment. Findings relating to sleep duration in BD in other studies have been mixed^37^, ^38^, ^39^ and we found that this may be due to measurement method: the BD group were more likely to have a longer sleep duration (more than 9 h) in both the objective and subjective measures compared to both UM and controls. However, the BD group were also more likely to report a short sleep duration (less than 7 h) in the subjective measure which was not supported by the objective measure. The BD group also experienced lower levels of sleep efficiency suggesting that they experience overall poor sleep quality. A follow‐up analysis comparing the difference between objective and subjective sleep duration across groups found that the UM group were more likely to *overestimate* their sleep duration than the BD or control group. Both mood disorder groups were also more likely to *underestimate* sleep duration compared to controls.

Sleep duration in UM has not been extensively studied, but loss of sleep is an important trigger for mania in BD,^40^ and increased sleep duration can contribute to improvements in manic symptoms.^41^ We found that short sleep duration was more likely in UM, whether self‐reported or objectively assessed. Although objective measures of sleep and activity had a similar pattern of negative outcomes for both mood disorder groups, *average activity levels* may represent a useful differentiator between UM and BD. The UM group demonstrated higher levels of average activity than both BD and controls. A more detailed investigation of temporal daily activity patterns could lead to specific markers of individuals at greater risk of BD or UM and help to target the most appropriate interventions.^42^

Whilst rest‐activity outcomes are usually assessed in relation to depressive or manic episodes, in this analysis the timings of episodes of mania and depression were not known for these groups and the subjective measures were not limited to any specific time‐period. This suggests that chronic changes to sleep and activity may persist regardless of proximity to an episode of mania and/or depression which has implications for future study design and disease management.

Dimensional approaches to understanding (hypo)mania in absence of depressive symptoms at a genetic level have suggested that mania and depression represent two distinct pathways,^43^ but little is known about UM at a behavioural level. The above findings suggest that negative sleep, activity and mental health outcomes appear to be transdiagnostic across BD and UM; however, by analysing a variety of these outcomes in a large non‐clinical population we have found evidence of key differences that may support UM being nosologically distinct. This supports further research into dimensional approaches to classification at a behavioural level.

We acknowledge some limitations to this work. The UK Biobank cohort are older, healthier and somewhat more affluent than the general population, so there may be issues relating to representativeness and our method of classifying BD and UM mood disorder categories was based on self‐report measures rather than formal clinical assessments. We acknowledge that these groups are not identical to unipolar mania or bipolar disorder as defined in the DSM or ICD classifications. The nature of the large data collections within UK Biobank was such that a formal diagnostic interview was not feasible. Our groups were therefore constructed as pragmatic proxies of diagnoses, making use of all the available self‐reported questionnaire data within the dataset.

There are varying levels of time between self‐report measures and accelerometer measures, as these were administered separately between 2013 and 2017. Further, the UM sample size was relatively small compared to BD and healthy controls and the UM group mostly satisfied criteria for hypomania rather than mania. However, the strengths of this study include the relatively large samples and the comprehensive phenotyping information that was available, including high quality objective (actigraph) measures of rest/activity rhythmicity.

## 5 CONCLUSION

We identified negative outcomes in mental health, activity and sleep in both BD and UM groups compared to controls. For most measures, the BD group had worse outcomes, perhaps suggesting that UM is a less severe subgroup of BD. However, there were some key differences between UM and BD groups that provide some support for UM as nosologically distinct, specifically: a much higher proportion of males; an early chronotype preference; significantly shorter objective sleep duration; and increased levels of activity. We conclude that these findings may have implications for the assessment, classification and treatment of patients who do not experience episodes of major depression but who do have a history of hypomania and/or mania.

## AUTHORS' CONTRIBUTIONS

NS, LL, BC, HW and DS designed the study. CAW derived the rhythmicity variables and NS derived the mood disorder classifications. NS analysed the data and drafted the manuscript. All authors contributed to the editing of the manuscript and have approved the final version.

## FUNDING INFORMATION

NS is funded by an MRC Precision Medicine Doctoral Training Programme Studentship (Sept 2019 – Apr 2023): “Predicting outcomes for bipolar disorder and major depression by integrating circadian, neuroimaging and genetic data within UK Biobank: a machine learning approach.” DJS acknowledges support from a Lister Institute Prize Fellowship and an MRC Mental Health Data Pathfinder Award (MC_PC_17217). LML is supported by a Royal College of Physicians of Edinburgh JMAS Sim Fellowship.

## CONFLICT OF INTEREST

None.

## ETHICS APPROVAL AND CONSENT TO PARTICIPATE

Participants who accepted the invitation to join the UK Biobank cohort provided written, informed consent and UK Biobank has generic ethical approval from the North West Multi‐centre Research Ethics Committee (ref 11/NW/03820). This work was conducted under UK Biobank project approvals 6553 (PI Smith) and 26209 (PI Wyse).

## CONSENT FOR PUBLICATION

Not applicable.

## Supporting information


Appendix S1

Table S1

Table S2

## Data Availability

There are restrictions prohibiting the provision of data in this manuscript. The data were obtained from a third party, UK Biobank, upon application. Interested parties can apply for data from UK Biobank directly, at http://www.ukbiobank.ac.uk. UK Biobank will consider data applications from bona fide researchers for health‐related research that is in the public interest. All derived variable and full results will be returned to UK Biobank.
